# Rapid Detection of Bacterial and Fungal Pathogens Using the T2MR versus Blood Culture in Patients with Severe COVID-19

**DOI:** 10.1128/spectrum.00140-22

**Published:** 2022-06-13

**Authors:** Tamara Seitz, Johannes Holbik, Julian Hind, Georg Gibas, Mario Karolyi, Erich Pawelka, Marianna Traugott, Christoph Wenisch, Alexander Zoufaly

**Affiliations:** a Department of Infectious Diseases and Tropical Medicine, Klinik Favoriten, Vienna, Austria; b Faculty of Medicine, Sigmund Freud University, Vienna, Austria; Institut Pasteur

**Keywords:** COVID-19, SARS-COV-2, bacteremia, candidemia, secondary infection, superinfection

## Abstract

A high rate of bacterial and fungal superinfections was reported in critically ill patients with COVID-19. However, diagnosis can be challenging. The aim of this study is to evaluate the sensitivity and the clinical utility of the point-of-care method T2 magnetic resonance (T2MR) with the gold standard: the blood culture. T2MR can potentially detect five different *Candida* species and six common bacteria (so-called “ESKAPE” pathogens including Escherichia coli, Staphylococcus aureus, Klebsiella pneumoniae, Acinet`obacter baumanii, Pseudomonas aeruginosa, and Enterococcus faecium). If superinfection was suspected in patients with COVID-19 admitted to the intensive care unit, blood culture and two panels of T2MR were performed. Eighty-five diagnostic bundles were performed in 60 patients in total. T2MR detected an ESKAPE pathogen in 9 out of 85 (10.6%) samples, compared to BC in 3 out of 85 (3.5%). A *Candida* species was detected in 7 of 85 (8.2%) samples of T2MR compared to 1 out of 85(1.2%) in blood culture. The mean time to positive test result in samples with concordant positive results was 4.5 h with T2MR and 52.5 h with blood culture. The additional use of T2MR enables a highly sensitive and rapid detection of ESKAPE and *Candida* pathogens.

**IMPORTANCE** Coronavirus disease 2019 (COVID-19) has led to a high number of deaths since the beginning of the pandemic worldwide. One of the reasons is the high number of bacterial and fungal superinfections in patients suffering from critical disease. However, diagnosis is often challenging. In this study we could show that the additional use of the culture-independent method T2MR did not only show a much higher detection rate of bacterial and fungal pathogens but also a significantly shorter time until detection and therapy change compared to the gold standard: the blood culture. The implementation of T2MRin the care of patients with severe course of COVID-19 might lead to an earlier sufficient antimicrobial therapy and as a result lower mortality and less use of broad-spectrum unnecessary therapy reducing the risk of resistance development.

## INTRODUCTION

Coronavirus disease 2019 (COVID-19), caused by severe acute respiratory syndrome coronavirus 2 (SARS-CoV-2), has led to a high number of deaths since the beginning of the pandemic worldwide. COVID-19 patients with severe ARDS admitted to an intensive care unit often require mechanical ventilation, broad spectrum antimicrobial therapy, parenteral nutrition, and immunomodulating therapy with steroids and other immunomodulatory drugs. Furthermore, severe SARS-CoV-2 pneumonia can result in an inflamed alveolar space, which provides an ideal environment for microbial growth (1). These are all risk factors for development of fungal or bacterial superinfection (2–4). Recent studies have shown that patients suffering from COVID-19 present with a reduced number of CD4 and CD8 cells (3) increasing the susceptibility for bacteremia and candidemia further. Patients with superinfections were shown to have a significantly higher mortality rate (4); however, frequently, the causative pathogen remained unknown (5).

One reason might be excessive usage of antimicrobial therapy (6) leading to a reduced detection rate of pathogens in culture (7). Another possible explanation (2) is the lack of screening for fungal infections, especially for *Candida* species, leading to underdiagnosis of this pathogen. Recent studies reported a significantly higher rate of candidemia in patients admitted to intensive care unit (ICU) with COVID-19 compared to patients admitted to ICU without COVID-19 (2, 7, 8).

Blood culture (BC) is regarded as the “gold standard” for the detection of microbial pathogens related to bacteremia and candidemia. Yet, this detection method has been shown to have a low sensitivity of 21%–71% for *Candida* species (9) and a sensitivity of 80%–96% for bacteria prior to initiation of antimicrobial therapy. Following initiation of antimicrobial therapy, the sensitivity drops (7). Another challenge with BC is the long duration from sample collection until a result is received (10). However, to combat the high mortality of bloodstream infections (BSI), especially candidemia (11, 12), the timely administration of appropriate antimicrobial agents is of vital importance. Furthermore, the rapid identification of the underlying species is crucial for guiding therapy since different *Candida* species have distinct susceptibility patterns.

To improve the diagnosis of candidemia, BC is often combined with other methods. Tests assessing the presence of and targeting fungal antigens or antibodies are commonly used for the diagnosis of invasive fungal diseases. For example, the cell walls of most pathogenic fungi contain Β-d-glucan (BDG), which can thus be used as a surrogate marker for fungal infections. The Fungitell Assay (Associates of Cape Cod) detects BDG with a sensitivity of 78–97% and a specificity of 88–100% (13–16). A problem associated with this test is the high rate of false positive results due to hemolysis, excess manipulation of the sample, exposure of the patient to gauze or other materials that contain glucans, as well as administration of certain therapeutics such as immunoglobulins, albumin, coagulation factors, or plasma protein factor (13). Positive Fungitell results should therefore always be confirmed with another method. Circulating levels of mannan or antibodies (CandidaAg) directed against this component of yeast cell walls can be used to detect invasive candidiasis in patients. This method however is hampered by the quick clearance of mannan from the serum. In a recently published retrospective study (17), an insufficient sensitivity of 52%–65% and high specificity of 98% was shown.

Furthermore, there are clinical scores to predict the probability of candidemia: the *Candida*-Colonization-Index (CCI) or corrected *Candida*-Colonization-Index (cCCI) (18) and *Candida* “predictive rule” (CPR) (19). The CCI is calculated by the ratio of the number of distinct nonblood body sites colonized by *Candida* spp. to the total number of body sites cultured. The cCCI is the ratio of the number of distinct body sites showing heavy growth (+++ or ≥10^5^) to the total number of body sites with *Candida* spp. growth. An index > 0.5 is significant for an increased risk of candidemia. The CPR is positive if the patient is in an ICU more than 4 days, a central venous catheter is present, or antibiotic therapy is given and at least 2 risk factors exist (total parenteral nutrition, hemodialysis, major surgery, pancreatitis, corticosteroids, immunosuppressants). If CPR is positive, the sensitivity for presence of candidemia is 50% and specificity is 83% (19).

T2MR is a promising method utilizing T2 magnetic resonance for the rapid detection of pathogens in whole blood samples. At present, three panels using this method are available, 1 for the detection of 5 different *Candida* species (T2CandidaPanel); 1 for 6 common bacteria (“ESKAPE” pathogens including Escherichia coli, Staphylococcus aureus, Klebsiella pneumoniae, Acinetobacter baumannii, Pseudomonas aeruginosa, and Enterococcus faecium) (T2Bacteria Panel); and 1 for detection of 13 common resistance genes (T2ResistancePanel) (mecA/mecC, CTX-M 14/15, AmpC, KPC, NDM, VIM & IMP, OXA-48, and vanA/vanB). This method can detect minimal amounts of intact target cells (1 CFU/mL) significantly faster than BC (20, 21). Results published by Mylonakis et al. (22) demonstrate that T2MR might be a superior to blood culture for monitoring BSI upon initiation of antimicrobial therapy.

T2CandidaPanel and T2BacteriaPanel are FDA approved and CE marked, while T2ResistancePanel is CE marked.

### Aims.

The aim of the current study is to compare sensitivity and specificity of T2CandidaPanel and T2BacteriaPanel with blood culture as the gold-standard in routine pathogen detection for the assessment of superinfection in patients with COVID-19. Time until result as well as frequency and duration until therapy change will be documented and compared.

## RESULTS

A total of 60 patients with COVID-19 admitted to ICU were included in the study. The patient characteristics and outcomes are shown in Table 1.

**TABLE 1:** Characteristics and outcomes of the study population^*a*^

Characteristics	All
No. of patients	60
Mean age (± SD)	58.7 years (11.6)
Female sex (%)	16 (26.7%)
Systemic corticosteroids (%)	60 (100%)
Antimicrobial therapy (%)	52 (86.6%)
Parenteral nutrition	54 (90%)
Central venous line (%)	55 (91.6%)
Invasive ventilation (%)	53 (88%)
28 days mortality	32 (53.3%)
ICU admission	60 (100%)
Mean time until ICU admission (± SD)	9.8 days (6.8)

a ICU, intensive care unit.

Eighty-five diagnostic bundles, including T2CandidaPanel, T2BacteriaPanel, and 2 × 2 BC, were performed in total. Up to four diagnostic bundles were performed in individual patients (73.4% of participants one, 15% two, 5% three, and 6.6% four diagnostic bundles).

Nine of 85 (10.6%) T2CandidaPanels and 7 of 85 (8.2%) T2BacteriaPanels displayed a positive result. In two cases, two bacterial pathogens were detected with the same test, in all other instances only one pathogen was detected if the test result was positive. Twenty-four of 85 BC (28.2%) showed pathogen growth. The most common pathogens detected by T2MR were E. faecium and C. albicans/tropicalis. The most common bacterial pathogens in the BC were S. epidermidis (Table 2).

**TABLE 2:** Number of detected pathogens in T2MR and BC^*a*^

Pathogens	T2MR (*n*)	BC (*n*)
E. coli	1	1
S. aureus	1	1
K. pneumoniae	2	1
A. baumannii	0	0
P. aeruginosa	2	0
E. faecium	3	0
S. epidermidis	0	13
S. hominis	0	4
*S. haemolyticus*	0	1
E. cloacae	0	1
*Cutibacterium* spp.	0	2
No. of ESKAPE spp. detected	9	3
No. of bacteria spp. detected	9	24
C. albicans/tropicalis	8	0
C. parapsilosis	1	1
C. glabrata/C. krusei	0	0
No. of *Candida* spp. detected	9	1

a T2MR, T2 magnetic resonance; BC, blood culture.

### Characteristic of detected pathogens.

The median time since ICU admission until detection of ESKAPE pathogen in T2MR were 5 days (range: 0–19 days) and *Candida* spp. 8 days (range: 0–25 days).

Twenty-four out of 85 BC showed pathogen growth out of which 19 (22.4%) were considered contaminants by the physician in charge (47.4% S. epidermidis, 31.6% S. hominis, 5.3% *S. capitis*, 5.3% *S. haemolyticus*, and 10.4% *Cutibacterium* spp.). Of the five pathogens detected in BC that were considered true infections, four originated in peripheral and central blood. In three of five (S. epidermidis, S. aureus, and C. parapsilosis), the time to positivity in central blood was 2 h less than peripheral blood; thus, a catheter-related BSI was suspected.

The clinical characteristics of the nine cases of candidemia are listed in Table 3.

**TABLE 3:** Clinical scores and growth of *Candida* in different sites in the nine cases of candidemia^*a*^

Diagnostic procedure	*Candida* spp. detected in T2MR only (*n* = 8)	*Candida* spp. detected in T2MR and BC (*n* = 1)
BDG		
Positive (%)	75%	100%
Negative (%)	25%	0
Missing (*n*)	4	0
CandidaAg		
Positive	50%	100%
Negative	50%	0%
Missing	4	0
Tracheal secretion		
*Candida* growth	100%	0%
No *Candida* growth	0%	100%
Not done	1	0
Urine		
*Candida* growth	66.7%	0%
No *Candida* growth	33.3%	100%
Not done	2	0
Wound		
*Candida* growth	100%	0%
No *Candida* growth	0%	100%
Not done	7	0
CCI		
Positive	83.3%	0%
Negative	16.7%	100%
Not done	0	0
cCCI		
Positive	66.7%	0%
Negative	33.3%	100%
Not done	2	0
CPR		
Positive	62.5%	100%
Negative	37.5%	0%
Not done	0	0

a BDG, Β-d-glucan; CCI, *Candida*-Colonization-Index; cCCI, corrected *Candida*-Colonization-Index; CPR, and *Candida* predictive rule.

In comparison, in the 51 patients without detected *Candida*, BDG was performed in 9.8% and CandidaAg in 7.8%. BDG showed a positive result in 20% and CandidaAg in 0%. CPR score was performed in all patients and positive in 50.9% (26 of 51). Data for CCI and cCCI were available in 76.9% and positive in 41% (16 of 39).

### Time until result using T2MR and BC.

**FIG 1:**
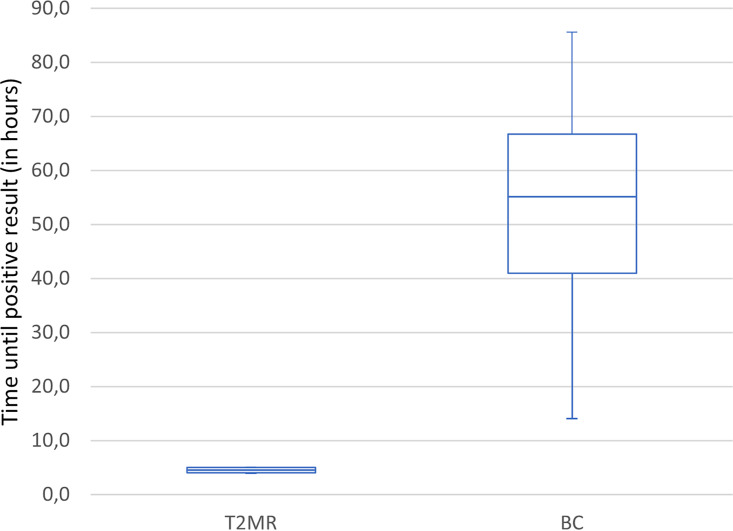
Difference of mean time until detection of all pathogens and confidence interval using T2 magnetic resonance (T2MR) and blood culture (BC).

In four samples, a concordant positive result in T2MR and BC (three samples with ESKAPE spp. and one sample with *Candida* spp. in T2MR and BC) was obtained. Mean time to positive test results was significantly shorter for T2MR (4.5 h) than for BC (52.5 h) as seen in Fig. 1.

The outcomes of these four concordant positive samples were compared using the *t* test. Since the number was very small, the Shapiro-Wilk test was performed to test for normality. The results of the *t* test showed a statistically significant difference (*P* < 0.05). The mean time until detection of ESKAPE spp. with T2BacteriaPanel was 4.3 h compared to 41.5 h with BC. The mean time until detection of *Candida* spp. with T2CandidaPanel was 5 h compared to 85.6 h with BC.

In samples with concordant negative results in T2MR and BC, mean time to negative test results for T2MR was 4.7 h compared to BC, which was 177.3 h. The difference was statistically significant (*P* < 0.05).

### Clinical relevance of T2MR.

Regarding ESKAPE pathogens and *Candida*, T2BacteriaPanel or T2CandidaPanel displayed a sensitivity of 100% compared to BC, given that *Candida* spp. or the ESKAPE pathogen was detected in BC, but not in T2MR. Regarding detection of all pathogens the sensitivity was 20% compared to BC. Only 28% (7 of 25) of BC results were considered true positive; therefore, sensitivity for T2MR regarding detection of all pathogens compared to BC increased to 71.4%. The results are summarized in Table 4.

**TABLE 4:** Sensitivity of T2MR compared to BC regarding detection of ESKAPE pathogens and all pathogens in blood

Sensitivity	T2MR
ESKAPE pathogens	100%
All pathogens (+ contamination)	20%
All pathogens causing infection	71.4%

The frequency of diagnostic bundle performance seems to influence positivity of T2MR, given that patients with one or two diagnostic bundles showed a positivity rate of 11.1–20.5% while 33%–50% of the patients receiving three or four diagnostic bundles had at least one positive result.

Antibiotic therapy was changed in 15.3% after T2MR results became available. The receival of result from BC led to a therapy adjustment in 2 of 85 cases (Table 5).

**TABLE 5:** Frequency and time until therapy change following result of T2MR or BC

Change of therapy	T2MR	BC
Therapy change	15.3% (13 of 85)	2.4% (2 of 85)
Escalation	77%	100%
De-escalation	23%	0%
Median time until therapy change (min-max, SD)		60.6 hours (95% CI: 376–497)
* *T2Candida	8 hours (95% CI 2.3–13.7)	
* *T2Bacteria	7.2 hours (95% CI 4.6–9.7)	

## DISCUSSION

The data of this study show that, compared to BC, T2MR enables a more rapid and sensitive detection of the most common bacterial pathogens as well as *Candida* spp. causing bloodstream infections in critically ill patients with COVID-19. In fact, without the additional use of T2MR, 13.3% of candidemia and 10% of bacterial superinfections would have been missed in our cohort.

Recent studies suggest that superinfections are common, but often underdiagnosed, in patients with severe case of COVID-19 (23). In a prospective study of 731 patients admitted to a hospital in Milan the overall 28-day cumulative incidence of superinfections was 16.4% (24). In a Chinese multicenter study including 476 COVID-19 patients, critically ill patients had the highest percentage of secondary bacterial infections (34.5%) compared to patients in the moderately ill and severely ill groups (3.9% and 8.3%), respectively (25) . In another study in Shanghai, only including critically ill COVID-19 patients admitted to ICU, a superinfection rate of 57% was described (26). Here, the most frequently detected pathogens were Gram-negative bacteria (50%), followed by Gram-positive bacteria (26%), viruses (11%), and fungi (7%) (26).

Superinfections were shown to be associated with a significantly worse outcome (4, 25). Therefore, the rapid detection of bacterial and fungal infection in COVID-19 patients admitted to ICU is of the utmost importance. However, such infections can be challenging to diagnose. In our study, 87% of the included patients had already received antimicrobial therapy at the time of blood collection. This may be one of the reasons for low detection rate of ESKAPE spp. in BC and the significantly higher sensitivity of T2MR. Antimicrobial therapy decreases the CFU of present microorganism to below levels of detection in BC, but not necessarily in T2MR, due to capability of detecting minimal amounts of intact target cells (1 CFU/mL),

The detection of candidemia is especially challenging. BC exhibits a low sensitivity (21–71%) (9) and, because of slow growth of this pathogen, it takes a long time until a result can be obtained. In our study *Candida* spp. was detected in BC in 1 of 60 patients compared to 9 out of 60 in T2MR, demonstrating the significantly higher sensitivity of T2MR. BDG and CandidaAg might be useful for diagnosis but are often not continuously available, like in our department. Positive *Candida* risk scores are a strong indicator for candidemia; however, their informative value might be limited in patients with severe COVID-19. For example, all patients with respiratory insufficiency receiving corticosteroids and the majority on the intensive care unit parenteral nutrition over a central line catheter. These patients would already fulfil the criteria for a positive CPR. In our cohort, patients with diagnosed candidemia had a positive CPR in 66.6% compared to 50.9% in patients without detected candidemia. CCI and cCCI was positive in 66.6% and 88.8% in patients with candidemia compared to 41% without.

Under any conditions, a sufficient candidemia diagnostic procedure is highly important, given that studies have shown a significantly higher rate of candidemia in patients admitted to ICU due to COVID-19 compared to patients without COVID-19 (7). Chen et al. performed a fungal culture in 99 COVID-19 patients with severe disease and detected *Candida* species in 4% (8). Yang et al. (2) reported that in 3 of 52 (5.8%) of patients admitted to the ICU a fungal coinfection was detected. The results of our study do not allow a statement about incidence. However, given that in 9 of 60 (15%) patients, in whom a superinfection was suspected, a *Candida* spp. was detected, a high rate can be suspected. The median time until detection of candidemia was 8 days following ICU admission and 15 days following admission to normal ward in our cohort. A large case-level analysis has similar results showing that median number of days since detection of candidemia and admission to normal ward was 14 days (27). In another study, candidemia was diagnosed at a median time of 16 days after ICU admission (28). Here, only BC was used, which could explain the longer time until detection compared to our cohort.

The use of T2MR in our cohort does not only enable a higher detection rate of ESKAPE and *Candida* spp. but also a significantly shortens the time until a result can be received (4.5 h versus 52.5 h). Duration until negative result was also significantly shorter. Based on the results of T2MR, therapy was modified in 15% of cases within our cohort. The time until appropriate therapy cannot be compared directly due to lack of randomization. However, the mean time until treatment modification after blood collection as a reaction to T2MR result was significantly reduced by more than 40 h when compared to the receival of result of BC. A shorter time until appropriate therapy is crucial, given that for every hour of delayed appropriate antimicrobial therapy during septic shock the survival rate decreases by 7.6% (29). In a quarter of the cases, an antibiotic de-escalation could also be achieved. Given that prolonged use of broad-spectrum antimicrobials is a known risk factor associated with the development and spread of antimicrobial-resistant organisms, this aspect demonstrates a further advantage of rapid pathogen detection.

It is important to underline that T2MR should only be used in conjunction with BC or samples from other sites (e.g., respiratory secretion) since T2MR can only detect a limited number of pathogens, only in blood, and culture offers the possibility for antibiotic resistance testing. To overcome this disadvantage, T2MR offers a T2ResistancePanel (30), which can detect antibiotic resistance, as well. However, this panel was not evaluated in this study.

A further disadvantage of T2MR is the much higher costs compared to BC for each test. However, by using T2MR as point-of-care method and therefore not requiring a professional laboratory and additional personnel, costs could be reduced. Furthermore, the higher detection rate of BSI and the shorter time until targeted therapy might have an impact in duration of hospital stay. This aspect must be addressed in future studies.

Our study has some limitations. First, there was no randomization between use of T2MR and BC; thus, the extent to which patients may directly benefit from a rapid pathogen detection and a decision to modify treatment could not be analyzed in this study. Further studies are necessary to address this gap. Second, there were no strict rules clarifying when to collect the diagnostic bundle and in many cases BDG or CandidaAg were not performed. On the other hand, this could also be regarded as a strong point given this reflects reality in a clinical setting.

Moreover, T2MR testing was performed directly after obtaining the blood samples by the treating physician in the setting of a specialized infectious diseases unit with attached point of care laboratory. This may limit the generalizability as the time to pathogen detection and time to treatment modification based on the results may be different and probably more comparable to BC in a setting where samples need to be transferred to a laboratory before analysis can be done. Even though a high number of samples were collected, the number of detected pathogens were low.

Nevertheless, to our knowledge, this is the first study, comparing T2MR with BC regarding the detection of secondary bacterial and fungal infections in COVID-19 patients.

In conclusion, the additional use of T2MR (T2CandidaPanel and T2BacteriaPanel) provides a highly sensitive and rapid detection of ESKAPE and *Candida* pathogens and therefore enables a shorter time duration until appropriate antimicrobial therapy administration compared to use of BC alone.

## MATERIALS AND METHODS

The study took place at the ICU of the Department of Infectious Diseases and Tropical Medicine, Clinic Favoriten, Vienna. During the time of the study, only patients suffering from severe COVID-19 were admitted. The study was approved by the local ethics committee (EK 20-790-VK).

### Procedure.

If superinfection (defined as a second infection superimposed by a different microbial agent than SARS-CoV-2) was suspected by the physician in charge, 10 mL of blood were drawn from the arterial catheter and/or central line for a diagnostic bundle (including 2 bottles for blood cultures and 2 EDTA tubes for T2CandidaPanel and T2BacteriaPanel). To include a patient in the study an infection with SARS-CoV-2 had to be confirmed (via PCR and naso-oropharyngeal swab), temperature must be ≥38°C or <36°C and clinical signs of bacterial or fungal infection (e.g., purulent sputum) and/or a rising of inflammation parameters, defined as rising of CRP or IL-6 or leukocyte count compared to previous day had to be present.

T2CandidaPanel and T2BacteriaPanel were performed immediately by the physician in charge at the point of care laboratory of the department. BC (BacT/Alert FN PLUS) bottles were sent to the microbiology department within 24 h at room temperature and then incubated following the company’s instructions (31).

The diagnostic bundle for suspected infections could be repeated as required.

Once the result of BC or T2Panel became available, therapy change was conducted at the discretion of the physician in charge. If a positive result was regarded as a contamination (e.g., by a skin commensal, due to inadequate blood collection), therapy remained unchanged. The physician in charge decided whether the result of BC and T2MR was classified as invalid due to contamination or true infection following the suggestions of CDC (32).

To confirm candidemia retrospectively, BDG, CandidaAg, and the risk scores CCI, cCCI, and CPR were performed. The cultivation of samples from different sites, e.g., respiratory secretion or urine was conducted at the discretion of the physician in charge.

### Statistical analyses.

Baseline parameters and outcome parameters of all included patients were collected. Test results of T2MR and BC were collected in a database and analyzed. Time of blood collection was documented and duration until result, frequency as well as time until therapy change of T2MR and BC was collected and compared. Therapy change was defined as de-escalation (change to a narrower spectrum antimicrobial therapy or cessation of antimicrobial therapy) or escalation (change to a broader antimicrobial therapy or initiation of therapy).

Sensitivity and specificity of T2CandidaPanel and T2BacteriaPanel compared to BC as the gold-standard were calculated. Furthermore, evidence for bloodstream infection as suggested by a positive result of T2MR or BC was ascertained by two infectious diseases physicians through reviewal of clinical records in individual cases. Sensitivities were recalculated using true clinical infection criteria as the comparator.

Continuous variables were summarized as mean and standard deviation and the groups were compared using the *t* test. Categorical variables between groups were compared using the chi-squared test. *P* < 0.05 was assumed as statistically significant. The calculations were performed with Graph Pad Prism (GraphPad Prism version 9.3.0 for macOS; GraphPad Software, San Diego, CA, USA; www.graphpad.com).
